# Quantitative ultrasound imaging of Achilles tendon integrity in symptomatic and asymptomatic individuals: reliability and minimal detectable change

**DOI:** 10.1186/s13047-016-0164-3

**Published:** 2016-08-17

**Authors:** Marie-Josée Nadeau, Amélie Desrochers, Martin Lamontagne, Christian Larivière, Dany H. Gagnon

**Affiliations:** 1 Pathokinesiology Laboratory, Centre for Interdisciplinary Research in Rehabilitation of Greater Montreal (CRIR) – Institut de réadaptation Lindsay-Gingras de Montréal, Centre intégré universitaire de santé et de services sociaux (CIUSSS) du Centre-Sud-de-l’Île-de-Montréal, Montreal, Canada; 2 School of Rehabilitation, Université de Montréal, Pavillon 7077 Avenue du Parc, Station Centre-Ville, P.O. Box 6128, Montreal, Quebec H3C 3J7 Canada; 3 Faculty of Medicine, Université de Montréal, Montreal, Canada; 4 Occupational Health and Safety Research Institute Robert-Sauvé (IRSST), Montreal, Canada

**Keywords:** Achilles tendon, Computer-assisted image analysis, Measures, Musculoskeletal system, Rehabilitation, Reproducibility, Tendinopathy, Ultrasonography, Quantitative evaluation

## Abstract

**Background:**

Quantifying the integrity of the Achilles tendon (AT) is a rehabilitation challenge. Adopting quantitative ultrasound measurements (QUS measurements) of the AT could fill this gap by 1) evaluating the test-retest reliability and accuracy of QUS measurements of the AT; 2) determining the best protocol for collecting QUS measurements in clinical practice.

**Methods:**

A total of 23 ATs with symptoms of Achilles tendinopathy and 63 asymptomatic ATs were evaluated. Eight images were recorded for each AT (2 visits × 2 evaluators × 2 images). Multiple sets of QUS measurements were taken: geometric (thickness, width, area), first-order statistics (computed from a grayscale histogram distribution: echogenicity, variance, skewness, kurtosis, entropy) and texture features (computed from co-occurrence matrices: contrast, energy, homogeneity). A generalizability study quantified the reliability and standard error of measurement (accuracy) of each QUS measurement, and a decision study identified the best measurement taking protocols.

**Results:**

Geometric QUS measurements demonstrated excellent accuracy and reliability. QUS measurements computed from the grayscale histogram distribution revealed poor accuracy and reliability. QUS measurements derived from co-occurrence matrices showed variable accuracy and moderate to excellent reliability. In clinical practice, using an average of the results of three images collected by a single evaluator during a single visit is recommended.

**Conclusions:**

The use of geometric QUS measurements enables quantification of AT integrity in clinical practice and research settings. More studies on QUS measurements derived from co-occurrence matrices are warranted.

## Background

The Achilles tendon (AT) is the largest and strongest tendon in the human body. The great tensile loads, occurring predominantly during its elongation or contraction, make it vulnerable to overuse injuries. Although the prevalence and incidence of midsubstance Achilles tendinopathy (i.e., in the middle third of the tendon) are high in athletes, cases are also frequently reported in sedentary individuals [1–6]. The aetiology and pathogenesis of AT tendinopathy have been the subject of much research, but with inconsistent findings [2, 3, 7]. Hence, treating people suffering from this pathology remains challenging for rehabilitation professionals and the success rate of conservative treatments is variable [8–10].

**Fig. 1:**
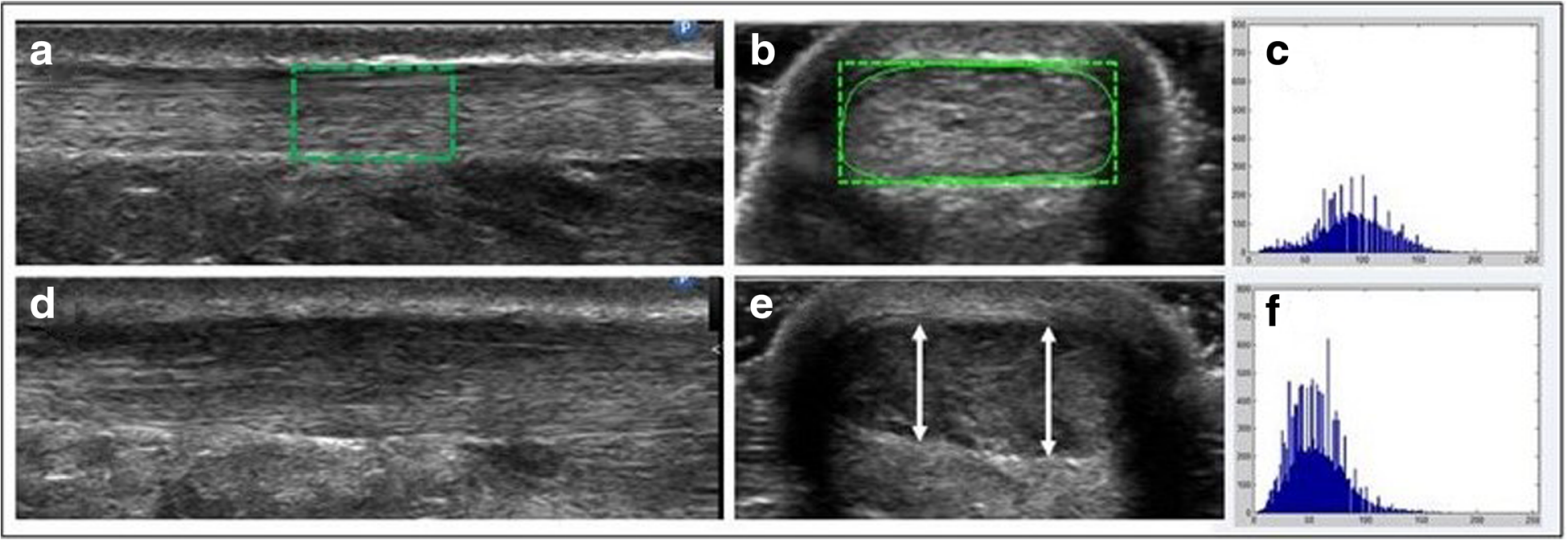
**a** ROI of a healthy AT in longitudinal view; **b** ROI of a healthy AT in transverse view; **c** grayscale histogram derived from the ROI of image (**b**); **d** Pathologic AT in longitudinal view; **e** Pathologic AT in transverse view with *arrows* indicating the AT’s thickness at different locations in the sagittal plane; **f** grayscale histogram derived from the ROI of image (**e**)

Ultrasound imaging allows in vivo visualization of the biological integrity of the tendon. It is a safe, rapid, non-invasive, relatively inexpensive and popular method used in the assessment of AT tendinopathy [11, 12]. When looking at ultrasound images (UIs) of healthy ATs, well-organized and parallel alignment of the collagen fibres (i.e., fibrillary striation) are highlighted by alternating parallel bright bands (hyperechoic) of collagen and dark bands (hypoechoic) of extracellular matrix [12, 13]. The paratenon of a healthy AT appears as an uninterrupted, well-defined bright line surrounding the tendon [12, 13] (Fig. 1). Conversely, in people with midsubstance AT tendinopathy, the fibrillar striation pattern is often altered as a result of a disorganization of the collagen fibres and a thickened and hypoechoic portion of the AT reflects an increase in the quantity of extracellular matrix and tenocytes [8, 14, 15]. This will typically translate to focal thickening along the AT, presence of dark (hypoechoic) intratendinous regions and sometimes irregular contours of the tendon on UIs [13] (Fig. 1).

Interpretation of an UI of the AT is generally semi-objective. The general appearance of the image is annotated based on the different contrasts observed (e.g., heterogeneous, homogenous, focal or diffuse abnormalities) and the maximum thickness of the AT is often measured using a two-point digital caliper function on the US machine. This interpretation is largely influenced by the evaluator’s experience with the recording technique and ability to interpret an UI [16, 17]. Recent technological advances have helped to promote the development of new quantitative ultrasound (QUS) outcome measures extracted from an UI, specifically from a particular region of interest (ROI). Digital UIs can now be broken down into a multitude of micro pixels, and numerical values (e.g., average thickness, tendon width and area) can be measured. The echogenicity of a ROI within an image can also be quantified by allocating a numerical grayscale value to each of those micro pixels [18, 19].

The usefulness of new UI analysis techniques has been demonstrated in various studies on animals and humans [20]. For example, these techniques have helped to quantify changes in the composition of an exercised muscle compared to an unexercised muscle in an elderly population [21–23]. These techniques have also revealed differences in the histological composition of the supraspinatus muscle and the quadriceps muscle in adults [24] and have been successfully used to detect structural changes in four key muscles in youths with neuromuscular disorders [25]. Moreover, new UI analysis techniques have enabled the differentiation of persons with Achilles tendinopathy from healthy individuals [26, 27] and have been effective in detecting focal and diffuse abnormalities in the AT [28].

Very few studies have been conducted to evaluate the reliability of QUS measurements of the AT. This is worrisome considering that the reliability of the QUS measurement of AT thickness, a key diagnostic criterion for Achilles tendinopathy, is rarely reported. To our knowledge, studies that have investigated test-retest reliability of QUS measurements of the AT have shown a moderate to good level of reliability [29–33]. In addition, it was shown that ultrasound image recording is greatly influenced by the evaluator, even among highly experienced ultrasonographers (weak inter-evaluator reliability [34]). Various factors such as the pressure applied on the probe and its alignment can influence recorded image properties and thus alter the quantitative values extracted [35, 36]. Information about the reliability and minimal detectable change is essential in order to develop evidence-based measurement taking protocols, empowering clinicians and researchers to quantify the tendinous changes observed in Achilles tendinopathy and incorporate these findings into clinical practice.

The primary objective of this study was to evaluate the reliability and minimal detectable change (MDC) of AT QUS measurements in people with symptoms consistent with midsubstance Achilles tendinopathy affecting at least one lower limb, as well as in completely asymptomatic individuals. The secondary objective was to recommend the best QUS measurement collection protocol possible, which could be subsequently used to characterize AT integrity in clinical practice or in research projects. It is anticipated that all QUS measurements, when collected by the same evaluator, will be reliable (Φ ≥0.75) and accurate (MDC_NORMALIZED_ ≤ 15 %) and that a QUS measurement taking protocol in which a single evaluator averages the results of at least three images obtained during a single visit will be recommended in clinical practice.

## Methods

### Participants

**Fig. 2:**
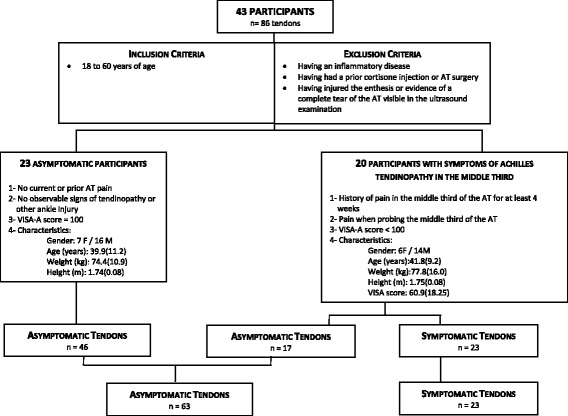
General criteria for inclusion and exclusion of participants and participants’ characteristics

A group of 20 individuals with clinical signs or symptoms of unilateral or bilateral midsubstance Achilles tendinopathy and a group of 23 asymptomatic individuals agreed to take part in this study. Individuals with symptoms consistent with Achilles tendinopathy had to have experienced pain over four weeks, evoked pain on palpation in the middle third of the AT and a VISA-A score below 100. The VISA-A questionnaire [37], completed by all the participants, is a reliable and validated measurement tool with an interest in AT pain, ability to function in daily life and during athletic activities. Eight questions are summed to produce an overall score, which is used as an indicator of the pathology’s severity. Scores range from 0 to 100, with a low score indicating greater severity. Asymptomatic participants were to have no pain or previous history of pain in the AT, no observable sign of Achilles tendinopathy or pain in the ankle, and a VISA-A score equal to 100 [38]. The criteria for the inclusion and exclusion of all participants, as well as each group’s specific characteristics, are summarized in Fig. 2. Finally, before conducting any formal testing, ultrasound visualization of the two ATs was performed for each participant to verify its integrity (i.e., normal tendon structure) at and around its insertion and also to rule out complete rupture of the AT. This experiment was approved by the Centre for Interdisciplinary Research in Rehabilitation of Greater Montreal (CRIR) Research Ethics Committee (Certificate: CRIR-557–1110). Participants were fully informed of the nature of the study and asked to sign a consent form before participating.

### Clinical examination

Initially, all the participants underwent a clinical examination conducted by an experienced physiotherapist specialized in musculoskeletal disorders with over 10 years of experience. This examination aimed at detecting signs and symptoms typically present with midsubstance Achilles tendinopathy. Special attention was given to the visualization of the AT (without ultrasonography), with emphasis on finding the characteristic thickening sometimes present in its middle third, as well as evoked pain on palpation of the AT’s middle third. A series of manoeuvres was carried out to apply passive and active tensions to selected structures with the intention of reproducing the participant’s symptoms at the AT: manually resisted contraction of the sural triceps, passive stretch of the sural triceps muscle, repeated unilateral heel rise test and repeated unilateral jump.

### Ultrasound image recording

#### Device and settings

All of the ultrasound examinations were conducted using a Philips HD11 1.0.6 ultrasound machine with a 5–12 MHz 50 mm linear array transducer (Philips Medical Systems, Bothell, WA). Image field depth was set to 2 cm, gain was set to 85 dB, probe frequency to 12 MHz and a unique focal zone (set at a depth of 0,5 cm) was positioned at the level of the AT. These main machine settings, as well as all the other options (e.g., compress, map, smooth, X-resolution) were maintained across all examinations performed for all participants in order to standardize the recorded images across all participants.

#### Ultrasonographers

A physiotherapist (M-J Nadeau) and a resident in physiatry (A. Desrochers) conducted all ultrasound examinations and recorded all of the AT images using a precise protocol (see next section). Both had previously received 10 h of practical training in AT ultrasound examination from an experienced physiatrist recognized by his peers in musculoskeletal ultrasonography (M. Lamontagne).

#### Image recording protocol

**Fig. 3:**
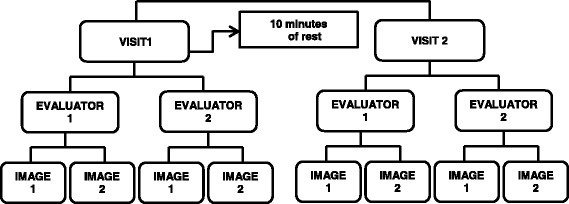
Summary of image recording protocol

A summary of the image recording protocol is illustrated in Fig. 3*. Initial visit (test)*: Each participant was placed in a prone position, with both feet dangling over the end of the table, and ankles positioned at about 5° of plantar flexion using a splint to immobilize the foot. Once placed in this position, the AT’s enthesis on the calcaneus was located by ultrasonography, and the skin marked at this location. The enthesis was defined as the most distal point of the insertion of the AT on the calcaneus. Another mark, made at a distance of 6 cm proximal to the enthesis, served as a standardized location for the center of the probe when performing the recording of all the ultrasound images. The images were recorded in this precise location since studies indicate that the incidence of Achilles tendinopathy is higher at this level (i.e., middle third) [2, 7]. The first evaluator recorded two images of the AT in the longitudinal view, as well as two additional images in the transverse view. During the recording of each of these images, the probe was removed and then repositioned on the skin with the center of the probe continually aligned with the mark on the skin. Once these four images were recorded, the first evaluator erased all the marks drawn on the skin before the second evaluator repeated the same image collection protocol. Particular attention was paid to the probe’s positioning on the tendon, taking care to apply minimal pressure on the probe and to align the transducer according to fiber orientation, with respect to the local referential (i.e., *x*, *y*, and *z* axis) defined by the tendon itself. Hence, the transducer was not necessarily perfectly aligned with the traditional anatomical planes and may have deviated slightly from them (i.e., yaw, roll and pitch movements of the transducer).

##### Second visit (retest)

After a minimal 10-min rest period, the evaluators repeated the image collection sequence described above. No significant change in QUS measurements was anticipated as each participant had to remain at rest between the two sessions.

### Image analysis

To calculate the different QUS measurements and facilitate characterization of the integrity of the AT, ultrasound images initially recorded in DICOM format were converted to JPEG format. An interactive 2D viewing and image analysis software, developed by the research team using MATLAB Image Processing Toolbox (The Mathworks, Natick, MA), was used to extract the QUS measurements. The development of this program was inspired by work previously realized by a research team based at the University of Pittsburgh that used QUS measurements to characterize shoulder tendons (i.e., supraspinatus, biceps) [39–41]. Each image selected for analysis appeared on screen and the evaluators (i.e., the physiotherapist and a trained research associate) traced a standardized ROI directly on the image, using markers. For blinding purposes, all images recorded during visits 1 and 2 by a unique evaluator were uploaded as a block of images prior to starting the image analysis. Thereafter, each image was presented in a random order to the evaluator to conduct the image analysis. While conducting the image analysis, only the image appeared on the computer screen and all other information was blinded with a black frame generated by the program. As described in detail below, the anatomical landmarks defining the ROIs in the longitudinal and transverse images of the AT differed between both views (Fig. 1a and b).

In the longitudinal view images, the ROI included a 1-cm length area, centered in the middle of the image and captured 6 cm proximal to the tendon enthesis (Fig. 1a). For transverse view images, the ROI was defined by the tendon’s contour (Fig. 1b). The ROI outline in both images was established to include the AT’s fibres and exclude the paratenon.

These two ROIs were used to extract the following QUS measurements selected for this study: thickness, width (only for transverse images), area (only for transverse images), echogenicity, variance, skewness, kurtosis, entropy, contrast, energy and homogeneity.

#### Thickness

The average thickness of the tendon’s ROI is calculated in the longitudinal view. One hundred equidistant points are plotted respectively on the upper and lower edges of the AT and the distance between each pair of points is calculated. The 100 distance measurements are then averaged and represent the thickness. In the transverse view, the thickness of the AT is determined by encompassing the tendon with a rectangle (Fig. 1b). The height of the rectangle reflects the tendon’s maximum thickness (Fig. 1e).

#### Width

The width is determined from the rectangle that encompasses the tendon, as defined above. The rectangle’s width reflects the tendon’s width (Fig. 1b).

#### Area

The tendon’s area corresponds to the area of the region delimited by the tendon’s outline.

To calculate the other QUS measurements, the ROI is fragmented into multiple micro pixels (micro pixel = 0,0057 mm^2^) by the software and a numerical grayscale colour value is allocated to each micro pixel. The grayscale is a scale of colors used in imagery that ranges from 0 = black to white = 255 for a total of 256 possible shades.

The micro pixels’ grayscale values included in the ROI are initially represented by a grey level frequency distribution curve found in the ROI (grayscale histogram) (Fig. 1c and f). The following first-order statistics can be calculated from this distribution curve: *echogenicity*, *variance, skewness*, *kurtosis* and *entropy.* Additional information on QUS measurements are provided in Table 1.

**Table 1:** Definitions of QUS measurements and their mathematical formulas

QUS measurements computed from a grayscale histogram		Mathematical formulas
First order statistics		
Echogenicity Mean ($\overline{\mathrm{x}}$)	Mean of grayscale values of micro pixels encompassed within the ROI (from 0 (black) to 255 (white) inclusively).	$\overline{\mathrm{x}} = {\displaystyle {\sum}_{\mathrm{x}=1}^{\mathrm{M}}}{\displaystyle {\sum}_{\mathrm{y}=1}^{\mathrm{N}}}\frac{\mathrm{I}\left(\mathrm{x},\mathrm{y}\right)}{\mathrm{M}\mathrm{N}}$
Variance (σ^2^)	Dispersion around the mean of the grayscale values of micro pixels encompassed within the ROI.	${\upsigma}^2=\frac{{\displaystyle {\sum}_{\mathrm{X}=1}^{\mathrm{M}}}{\displaystyle {\sum}_{\mathrm{y}-1}^{\mathrm{N}}}{\left\{\mathrm{I}\left(\mathrm{x},\mathrm{y}\right)-\overline{\mathrm{x}}\right\}}^2}{\mathrm{M}\mathrm{N}}$
Skewness (S_k_)	Reflects the asymmetry of the grey level frequency distribution curve around its mean. A high coefficient (in absolute value) translates in a shifted distribution relative to the mean, while a zero coefficient indicates a symmetric distribution. In a positively skewed distribution, pixels intensities are biased toward lower values (shifted distribution to the left). In a negatively skewed distribution, pixels intensities are biased toward higher values (shifted distribution to the right).	${\mathrm{S}}_{\mathrm{k}}=\frac{1}{\mathrm{M}\mathrm{N}}\frac{{\displaystyle {\sum}_{\mathrm{x}=1}^{\mathrm{M}}}{\displaystyle {\sum}_{\mathrm{y}=1}^{\mathrm{N}}}{\left\{\mathrm{I}\left(\mathrm{x},\mathrm{y}\right)-\overline{\mathrm{x}}\right\}}^3}{\upsigma^3}$
Kurtosis (K_t_)	Reflects the flatness of the grey level frequency distribution curve around its mean. A diffuse distribution will translate in a lower kurtosis value. Distribution concentrated around its mean will translate in a higher kurtosis value.	${\mathrm{K}}_{\mathrm{t}}=\frac{1}{\mathrm{M}\mathrm{N}}\frac{{\displaystyle {\sum}_{\mathrm{x}=1}^{\mathrm{M}}}{\displaystyle {\sum}_{\mathrm{y}=1}^{\mathrm{N}}}{\left\{\mathrm{I}\left(\mathrm{x},\mathrm{y}\right)-\overline{\mathrm{x}}\right\}}^4}{\upsigma^4}$
Entropy (*E*)	Reflects disorder in a ROI. It considers the number of grey levels in a ROI, and the proportions of each grey level. There is an increased entropy when multiple grey level values are present in the ROI. Vice-versa, entropy equals zero if an image has a single grey level value for all its micro pixels.	$\mathrm{E}=-{\displaystyle {\sum}_{\mathrm{i}=0}^{255}}{\displaystyle {\sum}_{\mathrm{j}=0}^{255}}\mathrm{p}\left(\mathrm{i},\mathrm{j}\right){ \log}_2\left(\mathrm{p}\left(\mathrm{i},\mathrm{j}\right)\right)$
QUS Measurements computed from a co-occurence matrix		
Texture parameters		
Contrast (*I*_*con*_)	Contrast measures the difference of intensity between the grey level values of neighboring micro pixels. There is a reduced contrast in a constant image with lesser local variations of the grey level intensities. On the contrary, contrast is higher in an image containing a large amount of local sudden variations in the values of grey level intensities.	${\mathrm{I}}_{\mathrm{con}}={\displaystyle {\sum}_{\mathrm{i}=0}^{255}}{\displaystyle {\sum}_{\mathrm{j}=1}^{255}}{\left|\mathrm{i}-\mathrm{j}\right|}^2\ \mathrm{p}\left(\mathrm{i},\mathrm{j}\right)$
Energy (*I*_*eng*_)	Energy is linked to the regularity and consistency of the patterns in an image. High energy is measured in a constant and steady picture. Vice-versa, low energy is found in an image in which the contacts of grey level values are diverse, uncoordinated and random.	${\mathrm{I}}_{\mathrm{eng}}={\displaystyle {\sum}_{\mathrm{i}=0}^{255}}{\displaystyle {\sum}_{\mathrm{j}=0}^{255}}\mathrm{p}{\left(\mathrm{i},\mathrm{j}\right)}^2$
Homogeneity (*I*_*hmg*_)	Homogeneity is increased in an image with a large number of pixels having the same grey level values, with little grayscale transition (i.e., increased when there is a large area of the same color).	${\mathrm{I}}_{\mathrm{hmg}}={\displaystyle {\sum}_{\mathrm{i}=0}^{255}}{\displaystyle {\sum}_{\mathrm{j}=0}^{255}}\frac{1}{1+{\left(\mathrm{i}-\mathrm{j}\right)}^2}\mathrm{p}\left(\mathrm{i},\mathrm{j}\right)$

I(x,y) denotes the grayscale intensity at the x,y coordonates in a ROI comprising M rows and N columns. p(i,j) represent the element of a grey-level co-occurrence matrix and denotes the probability that grayscale intensities i and j are adjacent

Next, a co-occurrence matrix is calculated. Texture analysis using a co-occurrence matrix is based on the repeated occurrence of a typical pixel configuration in the image’s ROI. It considers how many pairs of pixels with specific grayscale values and a specific predefined spatial relationship (distance and relative orientation angle) are present in an ROI. In this study, pairs of pixels were calculated in four directions (angles = 0°, 45°, 90° 135°) and a distance of 10 pixels was determined. The following texture indicator measurements are derived from this matrix: *contrast*, *energy* and *homogeneity* (Table 1).

All of the QUS measurements can be classified into three categories: *geometric measurements* (thickness, width, area), *measurements computed from a grayscale histogram* (echogenicity, variance, skewness, kurtosis, entropy) and *measurements computed from a co-occurrence matrix* (contrast, energy, homogeneity).

It is expected that a healthy tendon would have a more heterogeneous appearance because of the alternation of its black and white stripes, with a larger range of values on the grayscale. A pathological area in a tendon would, in contrast, have a darker, more homogeneous appearance, with grayscale values closer to zero (black). Hence, the following QUS measurement values are expected to be found in a pathological tendon: increased thickness, width, area, skewness, kurtosis, homogeneity and energy, as well as reduced echogenicity, variance, entropy and contrast [40].

### Statistical analysis

#### Outcome measures

The overall averages, standard deviations and confidence intervals of the results of the 8 images obtained for each QUS measurement in longitudinal and transverse views were calculated for all of the images for all tendons (*n =* 86) and separately for all of the images of symptomatic tendons (*n =* 23) and for all of the images of asymptomatic tendons (*n =* 63). The percentage difference between the averaged results of these two sub-groups was also calculated.

#### Reliability

The generalizability theory was used to determine the reliability of the different QUS measurements taken for the symptomatic and asymptomatic tendons. On the basis of the analysis of variance, this theory is mainly divided by 2 studies: the generalizability study (G-Study) and the dependability study (D-Study) [42]. Unlike the traditional theory of reliability that provides a unique random error term, the G-Study divides the error into different facets (sources of variance) relevant to our study and allows for the magnitude of the variance attributed to each facet to be determined. Therefore, in this study, the G-study determined the magnitude of the variance attributed to the subject (S), evaluator (E), visit (V), image (I), and random errors resulting from the interactions between these different sources of variance (SE, SV, SI, EV, EI, VI, SEV, SEI, SVI, EVI), thus leaving much less unexplained variance. In the G-Study, the variance component assigned to the subject (S) represents the difference between the subjects. This proportion of variance is error-free. The unexplained residual error is solely from the interaction between all sources of error and corresponds to the combination of the variances of subjects, evaluators, visits, and images (SEVI). Unlike the traditional theory of reliability that assumes that reliability exists independently of the measurement protocol design, the D-study relies upon information generated from the G-study to determine the reliability of specific simulated protocol designs and provides information to optimize reliability according to, for example, the context in which the measurement is being used (e.g., clinical practice versus research). In this study, the impact of different experimental protocols on the reliability coefficients (Φ), standard error of measurement (SEM) and normalized minimal detectable change (MDC_NORM)_ for each QUS measurement was determined. Since it is documented in studies that inter-rater reliability of QUS measurements is clearly inferior to intra-rater reliability, a single evaluator was used in the D-study. Improvements which may be obtained by averaging 1–3 images during a single visit or by averaging the images obtained during two visits by a single evaluator were compared. The G- and D-studies allow for the calculation of dependability coefficients (Φ), ranging from 0 (no reliability) to 1 (perfect reliability). In general, the dependability coefficient (Φ) can be interpreted as follows: poor reliability (Φ < 0.50), moderate (0.50 ≤ Φ < 0.75), good (0.75 ≤ Φ < 0.90) and excellent (Φ ≥ 0.90). However, there is some consensus that reliability indicators must exceed 0.90 for clinical measurements on an individual basis in order to minimize error and ensure reasonable validity [43]. More liberal reliability scores are allowed on a group basis, particularly for research purposes. The generalizability analysis was conducted using PC GENOVA statistical software, Version 2.2.

#### Standard error of measurement

Because the dependability coefficient (Φ) can be high despite substantial variability in the measurements, the standard error of measurement (SEM) has also been reported. The absolute SEM is estimated using the same units as the primary outcome measure. The SEM, which is the square root of the error variance, reflects the accuracy of a measurement.

#### Minimal detectable change

The absolute minimal detectable change (MDC_ABS_) was calculated to determine the extent of the absolute change required to detect a difference that could be interpreted as a real difference exceeding the measurement error. For a 90 % confidence level (*z* = 1.65), which is considered sufficient for clinical decision-making, the MDC_ABS_ was calculated using the following equation:

$$ \mathrm{M}\mathrm{D}{\mathrm{C}}_{\mathrm{ABS}} = 1.65\kern0.5em \times \kern0.5em \surd 2\kern0.5em \times \kern0.5em \mathrm{S}\mathrm{E}\mathrm{M} $$

In order for the MDC to be independent from the unit of measurement and to facilitate its interpretation, the MDC_ABS_ has been subsequently normalized relative to the average obtained (MDC_NORM_) and calculated using the following equation:

$$ \mathrm{M}\mathrm{D}{\mathrm{C}}_{\mathrm{NORM}} = \left(\mathrm{M}\mathrm{D}{\mathrm{C}}_{\mathrm{ABS}}\ /\ \mathrm{overall}\ \mathrm{group}\ \mathrm{average}\right) \times 100. $$

MDC_NORM_ ≤15 % reflects excellent measurement accuracy.

## Results

The overall averages, standard deviations and confidence intervals of the results of the 8 images obtained for each QUS measurement in longitudinal and transverse views for the complete set of tendons (*n =* 86) as well as for the symptomatic tendons (*n =* 23) and asymptomatic tendons (*n =* 63) are summarized in Table 2.

**Table 2:** Averages, standard deviations and confidence intervals of the different QUS measurements

Outcome measures (QUS Measurements)										
	All tendons *n* = 86			Symptomatic *n* = 23			Asymptomatic *n* = 63			Difference between groups (%)
	Average	Stand. Dev.	95 % CI^a^	Average	Stand. Dev.	95 % CI^a^	Average	Stand. Dev.	95 % CI^a^	
Longitudinal view										
Geometric										
Thickness (cm)	0.56	0.16	0.53–0.59	0.68	0.21	0.60–0.76	0.52	0.10	0.49–0.54	31.88
Grayscale histogram										
Echogenicity (0–255)	82.37	14.44	79.31–85.42	79.32	14.22	73.51–85.13	83.48	13.32	80.19–86.77	−4.98
Variance	618.72	216.98	572.86–664.58	654.93	213.52	567.66–742.19	605.50	160.62	565.83–645.16	8.16
Skewness	0.52	0.34	0.45–0.59	0.65	0.25	0.55–0.75	0.47	0.21	0.42–0.53	38.13
Kurtosis	3.82	1.28	3.54–4.09	4.18	1.02	3.76–4.59	3.68	0.64	3.52–3.84	13.41
Entropy	6.50	0.24	6.45–6.55	6.51	0.24	6.41–6.61	6.50	0.19	6.46–6.55	0.10
Co –occurrence matrix										
Contrast	0.64	0.13	0.61–0.67	0.61	0.11	0.57–0.66	0.65	0.11	0.62–0.68	−5.67
Energy	0.21	0.05	0.20–0.22	0.21	0.04	0.19–0.22	0.21	0.04	0.20–0.22	−2.76
Homogeneity	0.76	0.03	0.76–0.77	0.77	0.02	0.76–0.77	0.76	0.02	0.75–0.77	0.82
Transverse view										
Geometric										
Area (CSA) (cm^2^)	0.61	0.23	0.56–0.66	0.76	0.33	0.63–0.90	0.56	0.14	0.52–0.59	36.61
Thickness (cm)	0.57	0.15	0.54–0.60	0.67	0.20	0.59–0.75	0.53	0.09	0.51–0.55	26.73
Width (cm)	1.32	0.16	1.28–1.35	1.37	0.19	1.29–1.44	1.30	0.13	1.27–1.33	5.30
Grayscale histogram										
Echogenicity (0–255)	90.98	13.42	88.14–93.81	86.62	14.85	80.55–92.68	92.57	10.97	89.86–95.28	−6.43
Variance	767.57	207.20	723.77–811.36	789.63	180.44	715.88–863.37	759.51	150.15	722.44–796.59	3.96
Skewness	0.54	0.25	0.49–0.60	0.63	0.20	0.55–0.71	0.51	0.20	0.46–0.56	24.15
Kurtosis	3.61	0.83	3.43–3.78	3.78	0.75	3.48–4.09	3.55	0.58	3.40–3.69	6.68
Entropy	6.74	0.15	6.71–6.77	6.73	0.11	6.68–6.78	6.74	0.12	6.71–6.77	−0.15
Co-occurrence matrix										
Contrast	0.90	0.16	0.86–0.93	0.84	0.16	0.78–0.91	0.92	0.13	0.89–0.95	−8.33
Energy	0.14	0.04	0.13–0.15	0.15	0.03	0.13–0.16	0.14	0.03	0.13–0.14	5.76
Homogeneity	0.73	0.03	0.72–0.73	0.74	0.03	0.72–0.75	0.72	0.02	0.72–0.73	1.95

^a^Confidence intervals (CI) are calculated at a 95 % confidence level

### Sources of variance

The magnitude of each variance component (source of error), expressed as a percentage of the total variance for each QUS measurement for symptomatic and asymptomatic tendons, is presented in Tables 3 and 4 for images recorded in longitudinal and transverse views, respectively. Aside from the main source of variance associated with the subject (S) in most cases, the evaluator (E) is the systematic error with the highest variance percentage, up to 13.7 % of the total variance. The other systematic errors (visit and image) are negligible and vary from 0 to 1.9 % of the total variance. A significant proportion of random error is attributable to sources of variance that involve an interaction between the subject and the evaluator (SE, SEV, SEI) with proportions of up to 32.9 %, 40.0 %, and 16.3 % of the total variance, respectively. The contribution of the other errors (SV, SI, EV, EI VI, SVI, EVI) is lower, with percentages ranging from 0 to 10.8 %, where 10.8 % represents SVI interaction. The unexplained residual error (SEVI) is variable (1.5 to 27.3 %) for all of the measurements, with the exception of kurtosis (21.1 to 43.9 %) and skewness (22.7 to 39.2 %), which remains slightly higher.

**Table 3:** Magnitude of variance components for QUS measurements computed for the images recorded in longitudinal view

QUS measurements	Relative variance proportions for images recorded in longitudinal view (%)														
	S	E	V	I	SE	SV	SI	EV	EI	VI	SEV	SEI	SVI	EVI	SEVI
Geometric															
Thickness															
Sympt	83.0	2.2	0.0	0.0	0.0	0.0	0.0	1.0	0.0	0.0	6.2	4.0	1.7	0.0	1.9
Asympt	86.2	0.8	0.0	0.0	2.8	0.0	0.0	0.0	0.0	0.0	3.3	1.4	0.8	0.0	4.8
Grayscale histogram															
Echogenicity															
Sympt	75.7	0.0	0.0	0.0	7.7	0.0	0.0	0.0	0.0	0.1	8.8	1.2	0.5	0.0	5.8
Asympt	85.2	0.0	0.0	0.0	3.4	1.6	0.0	0.1	0.0	0.0	3.4	0.0	0.0	0.0	6.2
Variance															
Sympt	35.1	1.1	0.0	0.0	32.9	0.0	0.0	0.0	0.0	0.0	12.2	0.0	0.0	0.0	18.7
Asympt	61.7	0.0	0.1	0.0	1.0	0.0	0.0	0.0	0.0	0.0	8.7	0.0	1.3	0.0	27.3
Skewness															
Sympt	21.8	13.2	0.0	1.9	15.0	0.0	0.0	0.2	0.3	1.8	0.0	0.0	7.4	0.0	38.5
Asympt	26.8	7.6	0.0	0.0	12.7	0.0	0.0	0.0	0.4	0.1	7.3	11.0	3.9	0.0	30.2
Kurtosis															
Sympt	10.9	5.8	0.0	0.0	17.4	0.0	0.0	0.0	0.2	2.0	11.0	0.0	8.8	0.0	43.9
Asympt	16.3	7.3	0.0	0.0	19.1	0.5	0.3	0.0	1.1	0.0	9.3	13.4	1.9	0.0	30.8
Entropy															
Sympt	44.0	0.0	0.0	0.0	26.3	0.0	0.0	0.0	0.0	0.0	16.4	1.6	2.1	0.0	9.6
Asympt	58.8	0.2	0.2	0.0	5.7	0.0	0.0	0.0	0.0	0.0	10.2	0.0	5.5	0.0	19.5
Co-occurrence matrix															
Contrast															
Sympt	55.3	0.0	0.0	0.0	8.1	0.0	0.0	0.4	0.5	0.0	12.8	7.0	6.1	0.0	10.0
Asympt	61.3	0.0	0.0	0.0	4.5	0.8	0.0	0.9	0.0	0.2	5.1	0.0	4.2	0.0	22.9
Energy															
Sympt	40.9	0.0	0.0	0.4	19.6	0.0	0.4	0.0	0.0	0.0	17.8	0.9	0.0	0.0	20.0
Asympt	55.3	0.0	0.0	0.0	9.2	0.4	0.0	0.7	0.0	0.0	8.1	0.0	4.4	0.0	22.0
Homogeneity															
Sympt	56.3	0.0	0.0	0.0	6.3	0.0	0.0	0.0	0.0	0.0	12.5	4.7	7.8	0.0	12.5
Asympt	63.9	0.0	0.0	0.0	6.9	1.4	0.0	1.4	0.0	0.0	4.2	0.0	4.2	0.0	18.1

**Table 4:** Magnitude of variance components for QUS measurements computed for the images recorded in transverse view

QUS measurements	Relative variance proportions for images recorded in transverse view (%)														
	S	E	V	I	SE	SV	SI	EV	EI	VI	SEV	SEI	SVI	EVI	SEVI
Geometric															
Area (CSA)															
Sympt	92.8	0.0	0.0	0.0	3.0	0.1	0.2	0.0	0.0	0.1	0.9	0.0	0.4	0.0	2.4
Asympt	86.8	0.0	0.0	0.0	3.9	0.0	0.1	0.0	0.0	0.0	1.6	1.3	0.0	0.1	6.3
Thickness															
Sympt	92.6	0.0	0.0	0.0	3.3	0.2	0.1	0.0	0.0	0.0	1.9	0.4	0.0	0.1	1.5
Asympt	74.5	0.0	0.0	0.0	8.7	0.2	0.0	0.0	0.0	0.0	2.1	3.9	0.0	0.0	10.5
Width															
Sympt	80.4	0.0	0.0	0.0	2.0	0.0	0.2	0.0	0.0	0.4	2.1	0.0	0.0	0.0	15.0
Asympt	71.0	2.3	0.1	0.0	7.3	0.0	0.0	0.0	0.1	0.0	6.8	1.6	1.6	0.1	9.2
Grayscale histogram															
Echogenicity															
Sympt	81.3	4.4	0.0	0.0	0.0	0.0	0.0	0.0	0.0	0.1	6.1	1.5	1.5	0.4	4.6
Asympt	73.2	5.0	0.0	0.0	4.0	0.0	0.0	0.0	0.0	0.0	2.3	0.0	1.6	0.0	13.9
Variance															
Sympt	31.2	13.7	0.0	0.0	9.4	0.0	0.0	0.0	0.1	0.8	26.3	5.1	4.5	0.2	8.6
Asympt	49.9	10.1	0.0	0.0	6.7	1.4	0.0	0.0	0.0	0.0	10.7	2.8	2.0	0.0	16.5
Skewness															
Sympt	42.9	7.5	0.0	0.0	1.0	7.2	0.0	0.0	0.0	0.0	2.2	0.0	0.0	0.0	39.2
Asympt	58.9	0.0	0.0	0.0	0.8	1.0	0.1	0.0	0.2	0.0	9.0	7.0	0.0	0.3	22.7
Kurtosis															
Sympt	37.8	0.3	0.5	0.0	0.0	6.7	0.0	0.0	0.3	0.0	13.5	6.9	0.0	0.0	34.1
Asympt	44.6	0.7	0.0	0.0	0.0	0.0	0.0	0.0	0.0	0.0	11.8	16.3	4.7	0.8	21.1
Entropy															
Sympt	25.8	4.3	0.0	0.0	0.0	0.0	0.0	0.0	0.7	2.1	40.0	5.9	10.8	0.0	10.5
Asympt	48.8	3.6	0.0	0.0	12.3	4.2	3.6	0.0	0.2	0.2	5.7	1.8	0.0	0.0	19.5
Co-occurrence matrix															
Contrast															
Sympt	73.8	6.8	0.0	0.0	1.3	0.0	1.4	0.0	0.0	0.0	8.3	0.1	0.0	0.4	7.8
Asympt	59.6	2.2	0.0	0.6	4.0	3.0	0.0	0.0	0.0	0.6	5.2	3.3	2.5	0.0	19.0
Energy															
Sympt	58.2	5.5	0.0	0.0	0.0	0.0	0.0	0.0	0.0	1.2	21.8	3.0	2.4	0.6	7.3
Asympt	68.9	4.9	0.0	0.0	4.9	2.5	0.8	0.0	0.0	0.0	3.3	0.0	0.0	0.0	14.8
Homogeneity															
Sympt	80.8	6.1	0.0	0.0	0.0	0.0	0.0	0.0	0.0	0.0	4.0	0.0	0.0	1.0	8.1
Asympt	67.2	4.9	0.0	0.0	4.9	0.0	0.0	0.0	0.0	0.0	1.6	1.6	1.6	0.0	18.0

### Reliability and minimal detectable change

The reliability and MDC of different hypothetical QUS measurement acquisition protocol designs are described in Tables 5 and 6 for the images in longitudinal and transverse views, respectively. Different trends in reliability and MDC of the QUS measurements are observed for the three main measurement categories.

**Table 5:** Impact of different hypothetical protocols (D-Study) for QUS measurements recorded in longitudinal view

QUS measurements in longitudinal view			E = 1					
			V = 1			V = 2		
			I = 1	I = 2	I = 3	I = 1	I = 2	I = 3
Geometric								
Thickness	SYMPT	Φ	0.85	0.88	0.90	0.90	0.93	0.94
		SEM	0.09	0.07	0.07	0.07	0.06	0.05
		MDC90%_NORM_	29.21	25.17	23.66	23.28	19.35	17.84
	ASYMPT	Φ	0.90	0.93	0.94	0.94	0.96	0.97
		SEM	0.03	0.03	0.02	0.02	0.02	0.02
		MDC90%_NORM_	14.52	11.80	10.74	10.94	8.76	7.90
Grayscale histogram								
Echogenicity	SYMPT	Φ	0.83	0.87	0.88	0.90	0.93	0.93
		SEM	6.35	5.56	5.27	4.65	4.03	3.79
		MDC90%_NORM_	18.68	16.36	15.51	13.69	11.84	11.16
	ASYMPT	Φ	0.89	0.91	0.92	0.94	0.96	0.96
		SEM	4.76	4.05	3.78	3.37	2.87	2.68
		MDC90%_NORM_	13.31	11.32	10.57	9.43	8.01	7.48
Variance	SYMPT	Φ	0.69	0.76	0.79	0.81	0.86	0.88
		SEM	157.27	131.36	121.51	111.20	92.89	85.92
		MDC90%_NORM_	56.03	46.80	43.29	39.62	33.09	30.61
	ASYMPT	Φ	0.63	0.73	0.77	0.77	0.84	0.87
		SEM	118.97	93.45	83.22	84.12	66.08	58.85
		MDC90%_NORM_	45.85	36.01	32.07	32.42	25.47	22.68
Skewness	SYMPT	Φ	0.42	0.59	0.69	0.58	0.74	0.81
		SEM	0.29	0.21	0.17	0.21	0.15	0.12
		MDC90%_NORM_	104.67	74.18	60.70	75.60	53.58	43.84
	ASYMPT	Φ	0.43	0.57	0.64	0.55	0.69	0.75
		SEM	0.24	0.18	0.15	0.18	0.14	0.12
		MDC90%_NORM_	116.65	87.95	76.02	90.96	67.84	58.13
Kurtosis	SYMPT	Φ	0.30	0.42	0.49	0.46	0.59	0.66
		SEM	1.50	1.15	1.00	1.06	0.81	0.71
		MDC90%_NORM_	83.94	64.13	55.99	59.44	45.40	39.63
	ASYMPT	Φ	0.38	0.51	0.58	0.50	0.63	0.70
		SEM	0.81	0.62	0.54	0.64	0.48	0.42
		MDC90%_NORM_	51.31	39.27	34.34	40.69	30.67	26.51
Entropy	SYMPT	Φ	0.70	0.75	0.77	0.82	0.85	0.87
		SEM	0.17	0.15	0.14	0.12	0.10	0.10
		MDC90%_NORM_	5.92	5.21	4.95	4.30	3.75	3.55
	ASYMPT	Φ	0.65	0.74	0.77	0.78	0.85	0.87
		SEM	0.13	0.11	0.10	0.09	0.08	0.07
		MDC90%_NORM_	4.79	3.85	3.48	3.39	2.72	2.46
Co-occurrence matrix								
Contrast	SYMPT	Φ	0.63	0.72	0.75	0.74	0.82	0.84
		SEM	0.08	0.07	0.06	0.06	0.05	0.05
		MDC90%_NORM_	30.57	25.20	23.14	23.72	19.11	17.30
	ASYMPT	Φ	0.66	0.76	0.80	0.79	0.87	0.89
		SEM	0.08	0.06	0.05	0.06	0.04	0.04
		MDC90%_NORM_	28.23	21.88	19.30	19.96	15.47	13.65
Energy	SYMPT	Φ	0.60	0.68	0.71	0.75	0.80	0.83
		SEM	0.03	0.03	0.02	0.02	0.02	0.02
		MDC90%_NORM_	33.74	28.73	26.85	24.39	20.63	19.21
	ASYMPT	Φ	0.64	0.74	0.78	0.78	0.85	0.88
		SEM	0.03	0.02	0.02	0.02	0.02	0.02
		MDC90%_NORM_	34.26	27.17	24.35	24.22	19.21	17.22
Homogeneity	SYMPT	Φ	0.63	0.71	0.75	0.75	0.82	0.85
		SEM	0.02	0.01	0.01	0.01	0.01	0.01
		MDC90%_NORM_	4.72	3.85	3.52	3.54	2.85	2.58
	ASYMPT	Φ	0.71	0.80	0.83	0.83	0.89	0.91
		SEM	0.01	0.01	0.01	0.01	0.01	0.01
		MDC90%_NORM_	4.45	3.50	3.12	3.15	2.48	2.21

**Table 6:** Impact of different hypothetical protocols (D-Study) for QUS measurements recorded in transverse view

QUS measurements in transverse view			E = 1					
			V = 1			V = 2		
			I = 1	I = 2	I = 3	I = 1	I = 2	I = 3
Geometric								
Area (CSA)	SYMPT	Φ	0.96	0.97	0.98	0.98	0.99	0.99
		SEM	0.07	0.05	0.05	0.05	0.04	0.03
		MDC90%_NORM_	20.91	16.58	14.85	15.18	11.97	10.69
	ASYMPT	Φ	0.91	0.94	0.96	0.94	0.97	0.98
		SEM	0.05	0.03	0.03	0.03	0.03	0.02
		MDC90%_NORM_	18.96	14.50	12.67	14.38	10.89	9.45
Thickness	SYMPT	Φ	0.96	0.97	0.97	0.98	0.98	0.99
		SEM	0.04	0.04	0.03	0.03	0.03	0.03
		MDC90%_NORM_	14.79	12.85	12.13	11.02	9.41	8.81
	ASYMPT	Φ	0.83	0.90	0.92	0.89	0.94	0.95
		SEM	0.04	0.03	0.03	0.03	0.02	0.02
		MDC90%_NORM_	17.46	13.18	11.41	13.72	10.24	8.78
Width	SYMPT	Φ	0.82	0.89	0.92	0.90	0.94	0.96
		SEM	0.09	0.06	0.06	0.06	0.05	0.04
		MDC90%_NORM_	14.69	10.98	9.42	10.44	7.80	6.69
	ASYMPT	Φ	0.80	0.86	0.88	0.88	0.92	0.93
		SEM	0.06	0.05	0.05	0.05	0.04	0.04
		MDC90%_NORM_	11.61	9.55	8.75	8.56	6.97	6.35
Grayscale histogram								
Echogenicity	SYMPT	Φ	0.85	0.89	0.90	0.91	0.94	0.95
		SEM	6.11	5.16	4.81	4.55	3.79	3.50
		MDC90%_NORM_	16.47	13.91	12.95	12.25	10.20	9.42
	ASYMPT	Φ	0.81	0.88	0.91	0.90	0.94	0.95
		SEM	5.25	3.94	3.39	3.71	2.79	2.40
		MDC90%_NORM_	13.23	9.93	8.56	9.35	7.02	6.05
Variance	SYMPT	Φ	0.47	0.53	0.55	0.61	0.68	0.70
		SEM	179.83	159.58	152.23	134.27	116.89	110.49
		MDC90%_NORM_	53.14	47.16	44.99	39.68	34.54	32.65
	ASYMPT	Φ	0.63	0.71	0.75	0.76	0.82	0.85
		SEM	112.00	92.43	84.91	82.42	67.32	61.47
		MDC90%_NORM_	34.41	28.40	26.09	25.32	20.68	18.89
Skewness	SYMPT	Φ	0.47	0.60	0.66	0.64	0.75	0.80
		SEM	0.19	0.15	0.13	0.13	0.10	0.09
		MDC90%_NORM_	69.88	53.98	47.51	49.41	38.17	33.59
	ASYMPT	Φ	0.60	0.70	0.75	0.72	0.81	0.84
		SEM	0.15	0.12	0.11	0.12	0.09	0.08
		MDC90%_NORM_	69.85	55.19	49.35	53.67	41.75	36.94
Kurtosis	SYMPT	Φ	0.38	0.48	0.52	0.52	0.63	0.67
		SEM	0.83	0.68	0.62	0.62	0.50	0.45
		MDC90%_NORM_	51.01	41.66	38.04	38.09	30.71	27.81
	ASYMPT	Φ	0.45	0.57	0.63	0.56	0.68	0.74
		SEM	0.58	0.45	0.40	0.47	0.36	0.31
		MDC90%_NORM_	38.08	29.68	26.29	30.68	23.42	20.44
Entropy	SYMPT	Φ	0.27	0.32	0.34	0.40	0.47	0.50
		SEM	0.15	0.13	0.13	0.11	0.10	0.09
		MDC90%_NORM_	5.17	4.58	4.37	3.82	3.34	3.16
	ASYMPT	Φ	0.63	0.73	0.77	0.75	0.83	0.86
		SEM	0.09	0.07	0.06	0.07	0.05	0.05
		MDC90%_NORM_	3.02	2.42	2.18	2.30	1.81	1.62
Co-occurrence matrix								
Contrast	SYMPT	Φ	0.81	0.85	0.87	0.88	0.92	0.93
		SEM	0.08	0.07	0.06	0.06	0.05	0.04
		MDC90%_NORM_	21.25	18.16	17.01	15.64	13.20	12.29
	ASYMPT	Φ	0.65	0.75	0.79	0.77	0.85	0.88
		SEM	0.09	0.07	0.06	0.07	0.05	0.05
		MDC90%_NORM_	22.63	17.82	15.90	16.89	13.17	11.67
Energy	SYMPT	Φ	0.62	0.67	0.69	0.75	0.79	0.81
		SEM	0.02	0.02	0.02	0.02	0.02	0.02
		MDC90%_NORM_	39.31	35.16	33.67	28.93	25.50	24.25
	ASYMPT	Φ	0.78	0.85	0.87	0.87	0.91	0.93
		SEM	0.02	0.01	0.01	0.01	0.01	0.01
		MDC90%_NORM_	27.37	21.80	19.60	19.72	15.65	14.03
Homogeneity	SYMPT	Φ	0.86	0.90	0.92	0.92	0.95	0.96
		SEM	0.01	0.01	0.01	0.01	0.01	0.01
		MDC90%_NORM_	3.61	2.92	2.65	2.55	2.07	1.87
	ASYMPT	Φ	0.76	0.85	0.89	0.85	0.92	0.94
		SEM	0.01	0.01	0.01	0.01	0.01	0.01
		MDC90%_NORM_	3.82	2.80	2.36	2.80	2.04	1.72

The reliability and MDC of the results for the protocol design in which the QUS measurement results of three images taken by a single evaluator in a single visit (E = 1, V = 1, I = 3) are averaged were compared for the three main measurement categories. This measurement scenario is compatible with clinical practice.

#### Geometric measurements

In general, these QUS measurements have shown good to excellent reliability, with dependability coefficients ranging from 0.88 to 0.98 and good accuracy with a MDC 90%_NORM_ <15 % obtained in most cases. Only the thickness of symptomatic ATs in longitudinal view had a MDC 90%_NORM_ value greater than 15 % (MDC 90 % _NORM_ = 23.66 %) which still remains acceptable.

#### Measurements computed from a grayscale histogram

Echogenicity stands out in this category by its excellent results with dependability coefficients ranging from 0.88 to 0.92 and a MDC90%_NORM_ ranging from 8.56 to 15.51 %. The entropy also seems promising with a tendency for slightly better reliability than other QUS measurements in this category (all Φ =0.77 except for one Φ = 0.34), and an excellent MDC 90%_NORM_ ranging from 2.18 to 4.95 %. However, the other QUS measurements in this category have shown only weak to moderate reliability, with most dependability coefficients below the threshold of 0.75 (Φ range = 0.49–0.79). These measurements also showed a large MDC90%_NORM_ (with the exception of entropy) ranging from 26.09 to 76.02 %.

#### Measurements computed from a co-occurrence matrix

In general, these QUS measurements have shown moderate to excellent reliability with Φ ranging from Φ = 0.69 to Φ = 0.92 and a variable MDC90%_NORM_ ranging from 2.36 to 33.67 %. Of these measurements, homogeneity stands out with good to excellent reliability (Φ = 0.75 to Φ = 0.92) and excellent MDC 90%_NORM_ ranging from 2.36 to 3.52 %.

### Impact of averaging results from multiple images or visits

D-study reliability and error measurement estimates were computed for 6 experimental designs (Tables 5 and 6). Improved reliability and decreased MDC were obtained in all cases by increasing the number of recorded images. Slightly larger improvements in reliability and MDC were observed by recording images from two visits, in all cases.

## Discussion

### Evaluator as a significant source of variability

The evaluator represents a significant source of variability in the recording of UIs. The high level of technical skills and manual dexterity during UI recording requires extensive clinical experience and may contribute to variability associated with the evaluator [44]. In the present study, the limited experience of both ultrasonographers (i.e., evaluators) might have increased the variability of the evaluator (E) facet. However, Gellhorn et al. revealed excellent inter-rater reliability for the cross sectional area (CSA) measurement of the patellar tendon (comparable to the AT in terms of shape, content and superficial location) between a novice and an experienced sonographer [45]. The important interplay between the evaluator and the participants during an UI recording is highlighted by the high proportion of variance in all QUS measurements attributed to sources of error involving interaction between the subject and the evaluator (SE, SEV, SEI). Every AT is different and those anatomical and physiological differences (oblique orientation of the tendon, the subject’s weak natural echogenicity, blurred outline of the tendon, etc.) are expressed by in the subject facet (S). These dissimilarities make capturing an image of certain tendons more challenging than others and may explain why an evaluator may have more difficulty in assessing some subjects than others (SE). Consequently, it is recommended that a single evaluator record US images and extract QUS measurements, particularly when the goal is to monitor treatment effects over time.

### Superiority of geometric measurements and echogenicity

The excellent results obtained in this study, in terms of reliability and accuracy, of geometric QUS measurements of area, thickness and width (Φ obtained mostly at the top of the clinical acceptable threshold of 0.90 and a MDC90%_NORM_ <15 %) are similar or better than those obtained in comparable studies targeting the AT [31–33, 46]. Although echogenicity is a measurement computed from a grayscale histogram, it behaves as a geometric measurement and has also shown excellent reliability and accuracy (mostly all Φ > 0.90 and MDC_NORM_ < 15 %). Echogenicity has been previously studied, mainly on muscles, and has shown good reliability for repeated measurements (variation coefficients ranging from 5 to 11 %) [47–49]. Strict compliance of a standardized measurement taking protocol and the use of software to extract the geometric QUS measurements may explain, among other reasons, the favourable results obtained in this study. Continued use of the geometric QUS measurements, as well as echogenicity, is therefore encouraged in quantifying AT integrity.

The poor results, in terms of reliability and MDC, of the QUS measurements computed from a grayscale histogram (variance, skewness, kurtosis) obtained in this study confirm the need for refinement and further study before advocating the use of these QUS measurements in the assessment of AT integrity. For a hypothetical protocol in which the evaluator averages the results of three images recorded during a single visit (E = 1, V = 1, I = 3), the dependability coefficients obtained for variance, skewness and kurtosis range from 0.49 to 0.79, with the majority of measurements falling under the threshold established to ensure good reliability of 0.75. The accuracy results are also disappointing (MDC90%_NORM_ ranging from 26.09 to 76.02 %). Only entropy stands out for its general good reliability (all Φ =0.77 except for one Φ = 0.34) and high accuracy (MDC 90%_NORM_ ranging from 2.18 to 4.95 %) in this study when using the above-described protocol.

The reliability of QUS measurements computed from a grayscale histogram was also studied previously by two research teams that obtained similar results to those of the present study. Collinger et al. assessed the reliability and accuracy of various QUS measurements extracted from longitudinal images of the long head of the biceps and the supraspinatus tendons based on the generalizability theory [39]. The reliability and accuracy scores, determined with a D-study for an E = 1, V = 1, I = 2 protocol, are similar to the present study. Good reliability and accuracy of thickness (Φ ranging from 0.92 to 0.94; MDC90%_NORM_ ranging from 9.42 to 14.49 %) and echogenicity (Φ ranging from 0.79 to 0.85; MDC90%_NORM_ ranging from 16.03 to 18.72 %), as well as low reliability and weak accuracy of the variance, skewness and kurtosis have been found (Φ ranging from 0.57 to 0.69; MDC90% greater than 15 % and up to 297.35 %). Entropy demonstrated moderate reliability and good accuracy (Φ ranging from 0.64 to 0.68; MDC90% ranging from 5.56 to 5.58 %). Slightly better results in terms of reliability of the QUS measurements were obtained in the present study compared to the study by Collinger. The superior reliability of the AT QUS measurements is possibly explained by the fact that this tendon is easier to assess than shoulder tendons due to its superficial position, alignment and surrounding structures. Nielsen et al. assessed the test-retest reliability for repeated measurements of the QUS measurements of variance, skewness and kurtosis of the supraspinatus muscle. A single evaluator repeated 30 images during a single visit (E = 1, V = 1, I = 30) [49]. This team also obtained results consistent with those in this study, that is, low reliability in these QUS measurements, with variation coefficients (analogous to SEM expressed as a percentage of the grand mean) ranging from 14 to 35 %.

Knowledge of the theoretical foundations and the underlying calculation of the different QUS measurements computed from a grayscale histogram is essential in understanding the disappointing results, in terms of reliability and accuracy, of these measurements. The skewness and kurtosis QUS measurements are calculated directly from the shape of the grey level frequency distribution curve (grayscale histogram), while the shape of this curve also influences variance and entropy. For its part, the QUS measurement of echogenicity is an average of the grey scale values for all of the pixels in the ROI and does not take into account the shape of the distribution curve. The appearance of an anatomical structure on an ultrasound image can vary significantly depending upon the angle and the pressure applied to the tissues with the probe, both in terms of shape and echogenicity [50]. The edges of the ROI are sensitive to these variations in echogenicity, which can change the shape of the grey level frequency distribution curve without having a significant influence on the echogenicity’s average value for the ROI.

### Differences between transverse and longitudinal AT images

In this study, reliability and MDC of QUS measurements are generally similar between transverse and longitudinal images. The QUS measurement of thickness in a longitudinal view of symptomatic ATs is the exception to the rule as it tends to be less reliable (−8.58 %) and less accurate (−48.74 %) than in a transverse view. This difference might be explained by the fact that when the probe is positioned longitudinally to the AT fibres, it can be repositioned at different locations or angles on the tendon’s sagittal plane for each image (Fig. 1e). In a longitudinal view, the thickness measurement is captured only for a slice located directly under the probe and it is difficult to ensure that it is located on the thickest portion of the tendon. Therefore, it appears preferable that AT QUS thickness measurements are also taken in the transverse view, at a location considered relevant and determined following a full excursion of the transducer along the AT in both planes. Other thickness measures previously reported in the literature (e.g., true thickness measure) may also deserve to be explored in the future, especially with regard to the thickness measured in the transverse view [32]. In addition, since our QUS thickness measurement in the longitudinal view reflects the average thickness of a targeted area, it is likely that its value is reduced in comparison to the maximum thickness found in this region captured in the transverse view of the AT.

### Co-occurrence matrix shows promise in quantitative ultrasound imaging

The co-occurrence matrix is an image analysis method that considers the spatial organization of the pixels, as opposed to the grayscale histogram that only considers the grey scale values of the pixels, without taking into account their position on the image or their interaction with the surrounding pixels [18, 19, 51]. In our study, better reliability was achieved for QUS measurements drawn from a co-occurrence matrix in comparison to the reliability of the QUS measurements computed from a grayscale histogram. Collinger et al. obtained similar results [39]. Superior reliability may be explained by the fact that the co-occurrence matrix studies pairs of pixels, and not the isolated value of each pixel’s grey level.

### Proposing a measurement collection protocol for clinical practice

In clinical practice, it is difficult to consider having more than one assessment visit in which additional images would be recorded (V = 2). Even though one or more additional visits positively influences the reliability and accuracy of QUS measurements, productivity constraints should be considered. A protocol in which the averaged result obtained from three images collected by a unique evaluator during a single visit seems to represent a good compromise. The clinical applicability of AT QUS measurements becomes highly relevant since the time required for recording them is, in the latter protocol, at most 10 min, which can help in the clinical decision making process and in practice.

### Study limitations

This study has several limitations. Within the context of this reliability study, all measurements were taken in an identical location across all participants according to a standardized protocol. The location selected was set at 6 cm proximal to the enthesis of the AT considering that AT tendinopathy typically occurs between 2–6 cm proximal to its enthesis on the calcaneus [52, 53]. Hence, measurements of the symptomatic tendons were not necessarily done exactly at the pathology’s precise location for all participants which, in turn, may have minimized the variance between the pathological tendons and underestimated the reliability and accuracy of the measurements. Two separate tasks, which are both dependent upon the evaluator, must be performed when obtaining QUS measurements. The first task involves obtaining the ultrasound image (image acquiring) and the second consists of processing the acquired image in order to extract the desired quantitative values (image analyzing). Both “image acquiring” and “image analyzing” can affect reliability separately. For example, when the evaluator plots the delineation of the ROI while analyzing the UIs, the sometimes-blurred outline of the AT increases this task’s difficulty. Syha et al. found that reliability of thickness measurements of the AT was more reliable when the ROI was traced automatically compared to manual tracing [54, 55]. In the present study, it is impossible to differentiate the error related to the recording of the image from that of the analysis of the image. Further studies are required to isolate these potential sources of variability that are currently encompassed within the evaluator facet. Another source of variability and potential limitation of the study is that we are quantifying the integrity of a three dimensional tendon using two dimensional UIs.

## Conclusions

This study focused on the reliability of three types of QUS measurements: geometric QUS measurements, QUS measurements computed from a grayscale histogram, and QUS measurements computed from a co-occurrence matrix. Even though additional validity and responsiveness studies are necessary, the favourable results of geometric QUS measurements and of the echogenicity further support their use in clinical practice and research protocols. These measurements could be used in longitudinal follow-up to capture the progress of the severity of AT tendinopathy and the impact of follow-up treatment in clinical practice or in rehabilitation research protocols. These measurements may also be useful in a transversal context in order to compare individuals between themselves or against standards established in clinical practice or in future studies. Furthermore, it is imperative that particular emphasis be given to adhering to a rigorous, standardized measurement-taking protocol when acquiring UIs to reach an acceptable level of reliability and accuracy. With respect to QUS measurements computed from grayscale histograms, in light of the results obtained in this study, the use of these QUS measurements in evaluating AT integrity should be reconsidered. Lastly, QUS measurements computed from a co-occurrence matrix are promising and additional studies on this emerging method of image analysis are necessary.

## Abbreviations

ASYMPT: Asymptomatic
AT: Achilles tendon
CRIR: Centre for Interdisciplinary Research in Rehabilitation of Greater Montreal
CSA: Cross sectional area
E: Evaluator
I: Image
MDC: Minimal detectable change
MDC_ABS_: Absolute minimal detectable change
MDC_NORM_: Normalized minimal detectable change
QUS: Quantitative ultrasound
ROI: Region of interest
S: Subject
SEM: Standard error of measurement
SYMPT: Symptomatic
UI: Ultrasound image
V: Visit
VISA-A: Victorian Institute of Sport Assessment- Achilles
